# Unraveling the link between language barriers and cancer risk

**DOI:** 10.1007/s10552-024-01946-5

**Published:** 2024-12-11

**Authors:** Eman A. Toraih, Mohammad H. Hussein, Manal S. Malik, Alaa N. Malik, Emad Kandil, Manal S. Fawzy

**Affiliations:** 1 Department of Surgery, School of Medicine, Tulane University, New Orleans, LA 70112 USA; 2 Genetics Unit, Department of Histology and Cell Biology, Faculty of Medicine, Suez Canal University, Ismailia, 41522 Egypt; 3 Ochsner Clinic Foundation, New Orleans, LA USA; 4 School of Medicine, Tulane University, New Orleans, LA 70112 USA; 5 School of Medicine, Louisiana State University Health Sciences Center (LSUHSC), New Orleans, LA 70112 USA; 6 Center for Health Research, Northern Border University, 91431 Arar, Saudi Arabia

**Keywords:** Cancer, Language isolation, Language barriers, Risk, Outcomes

## Abstract

**Purpose:**

Clear patient communication with the physician is an integral aspect of cancer treatment and successful health outcomes. Previous research has shown improved cancer screening in cases of patient navigator assistance to limited English proficient patients, but no research has analyzed the relationship between language isolation and cancer incidence rates in the United States.

**Methods:**

Using state-level data from the United States Census Bureau and the National Cancer Institute, we analyzed the correlations between language isolation and age-adjusted incidence rates across 19 different invasive cancers.

**Results:**

A complex relationship between language isolation and cancer incidence rates was found. States such as California, New York, Texas, and New Jersey show high language isolate prevalence and elevated cancer incidence rates. Cancer subtype incidence rates varied between states, indicating the multifactorial importance of lifestyle, genetics, and environment in cancer. California had the highest language isolation ranking of 8.5% and elevated rates of ovarian (10.4/100,000) and stomach (9.1/100,000) cancers. New York, with the second-highest language isolation ranking of 7.6%, manifests a pronounced prevalence of ovarian (11.3/100,000) and stomach (10.9/100,000) cancers. Overall, positive correlations were observed between language isolation and ovarian/stomach cancers, while negative correlations were found with lung, kidney, melanoma, and colorectal cancers.

**Conclusion:**

This study emphasizes the need to address language barriers and other social determinants of health in cancer prevention/control. Targeted interventions, such as culturally appropriate education, increased access to linguistically and culturally appropriate cancer screening, and language lessons, are crucial in improving health outcomes in linguistically diverse communities.

**Supplementary Information:**

The online version contains supplementary material available at 10.1007/s10552-024-01946-5.

## Introduction

Cancer is a complex and multifactorial disease, with many risk factors contributing to its development [1]. While genetic predisposition and lifestyle choices are well-established risk factors, recent research has begun to investigate the impact of social and environmental factors on cancer risk [2]. The exposome concept has emerged as a framework for understanding the complex interplay of social, environmental, and biological factors contributing to cancer development [3]. One such factor that has gained attention in recent years is linguistic isolation, which refers to a situation where individuals have limited proficiency in the language spoken by most of the population in their region [3, 4].

The United States is a veritable melting pot of cultures and languages. According to the US Census Bureau, Spanish represents the second most commonly spoken language, with over 41 million speakers nationwide. Beyond English and Spanish, the diverse American populace speaks more than 350 languages within households, ranging from Pennsylvania Dutch, Ukrainian, and Turkish to Romanian, along with 150 indigenous North American languages such as Dakota, Apache, and Cherokee. While this diversity constitutes a formidable strength, it poses challenges for individuals who do not speak English [5]. These individuals may experience language isolation, a significant issue in the United States, where millions speak languages other than English at home. Language isolation engenders communication barriers, restricted access to health information and services, and ultimately, an elevated risk for many health conditions, including cancer [5–7].

The correlation between language isolation and cancer risk is intricate and multifarious, and the role of the exposome in this association has not been thoroughly investigated. Clear communication is integral to cancer prognosis, treatment, and follow-up care, but language barriers limit such communication and patient-centered care [6, 8, 9]. Patients from linguistically isolated communities lack access to crucial information about the importance of cancer screening, possess limited knowledge of screening guidelines, and face restricted access to cancer screening tests, resulting in suboptimal health outcomes [10, 11]. Lack of clear communication increases patient anxiety and reluctance to request additional information about their condition [9, 12, 13]. Research has demonstrated that language barriers contribute to lower screening rates for female-specific cancers requiring patient cooperation, such as pap smears and breast exams, ultimately resulting in delayed diagnosis and treatment, poor compliance with therapy, augmenting the risk of advanced cancer and other unfavorable outcomes in patients with language barriers [5, 14, 15].

The prevalence of non-English language isolation fluctuates across the United States. The US Census Bureau reveals that California, Texas, New York, Florida, and New Jersey harbor the highest percentage of non-English speakers. Consequently, it is crucial to explore the ramifications of language barriers on the incidence rates of various cancers among linguistically diverse patients to discern patterns and correlations that may unveil crucial insights into the epidemiology of these malignancies. In the current study, we investigated the correlation between linguistic isolation and cancer risk. This paper aims to provide valuable insights into the impact of language barriers on cancer risk, highlighting the need for targeted interventions to address this pressing public health issue and promote better health outcomes for these vulnerable populations.

## Methods

### Study design and data sources

This cross-sectional study analyzed two primary datasets: language isolation data from the U.S. Census Bureau (2017–2021) and cancer incidence data from the Surveillance, Epidemiology, and End Results (SEER) database (2001–2019). The investigation encompassed all 50 U.S. states.

### Language isolation assessment

Data on language isolation were obtained from the Census Bureau (http://www.census.gov/) and American Community Survey (http://www.census.gov/acs/www/). Language isolation was defined using a binary classification system based on strict Census Bureau criteria: households were categorized as language-isolated if no member aged 14 or older either (a) spoke only English or (b) spoke English “very well” while using another language. This classification, while enabling population-level analysis, has inherent limitations in capturing the spectrum of language proficiency and healthcare communication needs. The study population included all racial and ethnic groups, including Hispanics.

### Cancer incidence data collection

Cancer incidence data were extracted from the SEER program database, which covers approximately 28% of the U.S. population and provides comprehensive cancer incidence and survival data. The analysis included age-adjusted incidence rates for 19 cancer types: bladder, brain, breast, cervix, colorectal, esophagus, kidney, leukemia, liver, lung, melanoma, non-Hodgkin lymphoma, oral, ovary, pancreas, prostate, stomach, thyroid, and uterus. Age adjustment was performed using the 2000 U.S. standard population, with rates expressed per 100,000 individuals annually. For bladder cancer, both invasive and in situ cases were included; for all other cancers, only invasive cases were analyzed. SEER*Stat software was utilized for data extraction and initial processing.

### Trend analysis

The Average Annual Percent Change (AAPC) was calculated using the Joinpoint Regression Program. Trends were classified as: (1) ascending: when the 95% confidence interval (CI) of AAPC exceeded 0; (2) stable: 95% CI of AAPC includes 0, or (3) descending: 95% CI of AAPC was below 0. Data suppression was implemented where necessary to maintain confidentiality and statistical stability.

### Geospatial analysis

Cartographic visualization was performed to display state-level, age-adjusted incidence rates for all cancer types. These maps were created to identify geographic patterns and potential areas requiring targeted interventions.

### Statistical analysis

Data assessment was executed utilizing SPSS version 27.0. Tabular presentations were generated to exhibit the state-level cancer incidence rates, stratified by cancer type, and language isolation prevalence. Spearman's correlation analysis was employed to evaluate associations between the proportion of non-English language householders and cancer incidence rates for distinct cancer types. A correlation matrix and regression plots were generated. A p-value of less than 0.05 was set as the threshold for statistical significance.

## Results

### Geographic distribution of language isolation

**Fig. 1:**
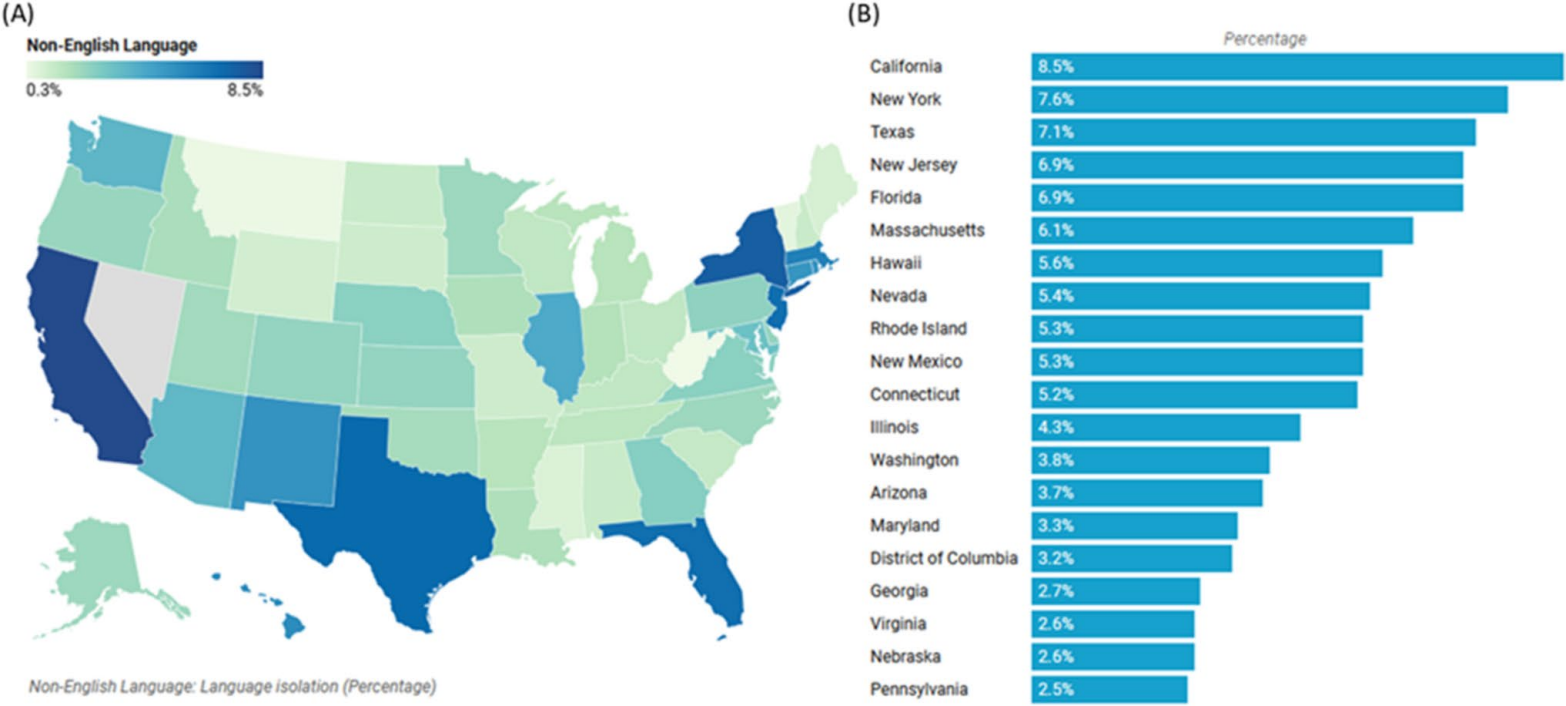
Prevalence of language isolation communities by State. Data is represented as a percentage of non-English language households within the State. **A** The United States map represents 5-year data for non-English Language Isolation for ages 14 + , all races (including Hispanics), and both sexes. **B** The bar chart represents the top 20 States with a higher prevalence of non-English households. A “limited English-speaking household” resident was defined as if there is no member 14 years old and over who (i) speaks only English or (ii) speaks a non-English language and speaks English "very well. "Data source: Demographic data for the United States by State 2017–2021 provided by the Census Bureau (http://www.census.gov/) & the American Community Survey (http://www.census.gov/acs/www/). Data for the United States does not include Puerto Rico. Created by statecancerprofiles.cancer.gov

Our analysis revealed that 5,241,326 households in the United States experience language isolation, accounting for 4.2% of all households. As depicted in Fig. 1, California leads with 8.5% language-isolated households, followed by New York (7.6%), Texas (7.1%), New Jersey and Florida (6.9% each). States with the lowest rates—Maine (0.9%), Mississippi (0.8%), Vermont (0.6%), Montana (0.4%), and West Virginia (0.3%)—share distinct demographic characteristics: predominantly rural populations (> 60% in Montana/Vermont), minimal immigration (< 5% foreign-born), limited urban centers, and high native-born populations (> 95%, exceeding national average).

### Cancer incidence

#### Overall cancer incidence patterns

**Fig. 2:**
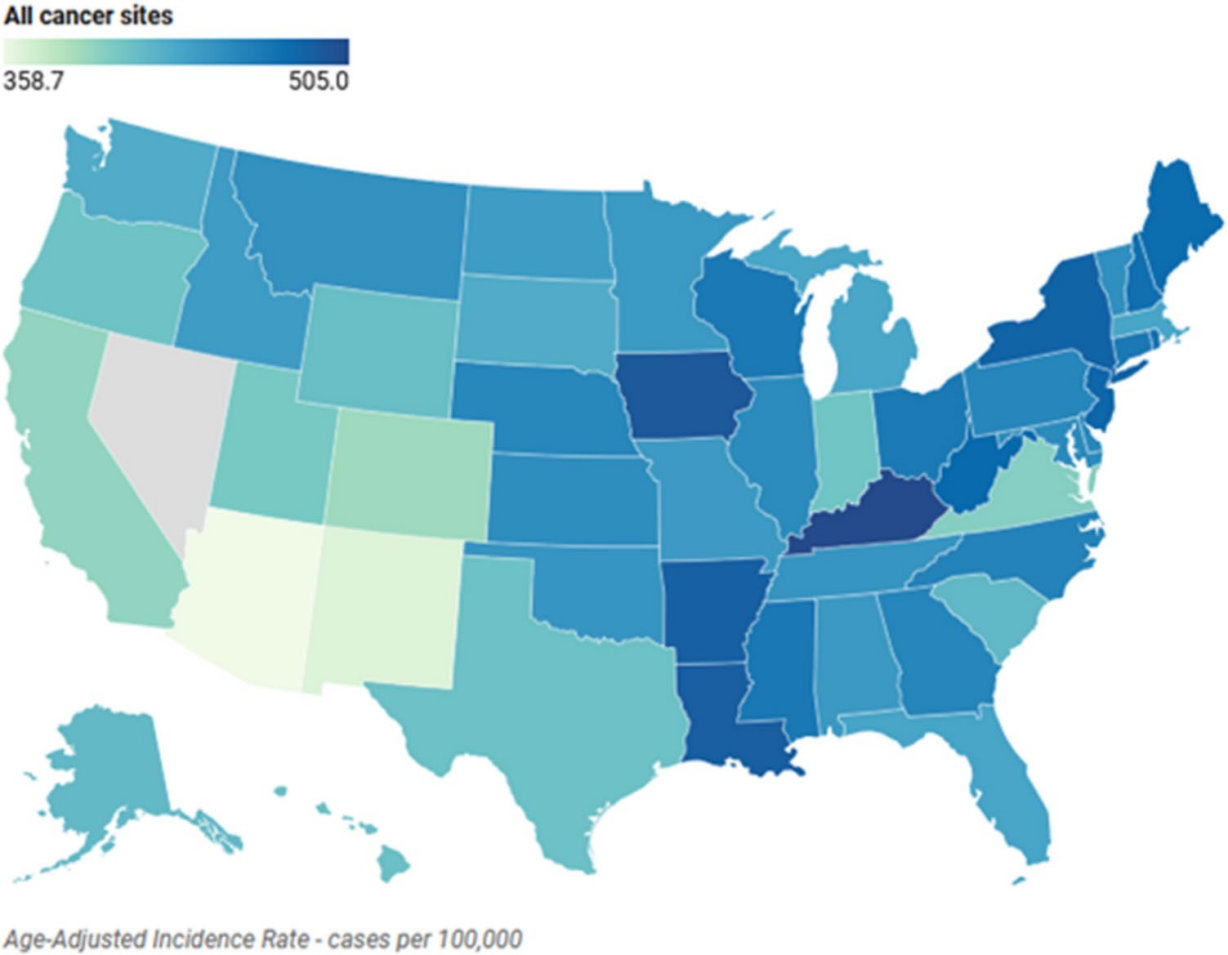
Overall cancer incidence rates across states. The incidence rates of prevalent cancers (all stages) were age-adjusted to the 2000 US standard population and were reported as cases per 100,000 population per year. Some data may be unavailable for certain combinations of geography, cancer site, age, and race/ethnicity and has been suppressed to ensure the confidentiality and stability of rate estimates. Data source: Data from the National Program of Cancer Registries and SEER*Stat Database (2001–2019) (https://ephtracking.cdc.gov/DataExplorer/)

Cancer incidence rates vary significantly across states (Fig. 2), with Kentucky reporting the highest rate (505 per 100,000), followed by Iowa (494.1) and Louisiana (489.2), while Arizona (358.7) and New Mexico (366.5) show the lowest rates. Supplementary Table S1 details incidence rates for 19 cancer types across all states.

#### Gender-specific cancer distribution

Breast cancer shows the highest overall incidence nationwide (114.9–147.2/100,000), peaking in Delaware and lowest in Wyoming. Prostate cancer demonstrates wide variation, with Florida reporting the lowest rate (88.2/100,000) and Louisiana the highest (146.5/100,000). Cervical cancer rates vary substantially, from Kentucky's high of 9.2/100,000 to Massachusetts’ low of 4.8/100,000.

#### Regional cancer patterns

Northern states exhibit distinctly higher melanoma rates, particularly in Vermont (32.5/100,000), Minnesota (29.0/100,000), and surrounding states. Kentucky leads in both lung (75.6/100,000) and colorectal (40.3/100,000) cancer rates, while the Northeast shows elevated stomach cancer rates, notably in New York (10.9/100,000) and New Jersey (9.3/100,000).

#### State-specific cancer findings

Several states show unique cancer patterns: Alaska reports exceptionally high esophageal cancer rates (15.0/100,000 versus typical 1.2–2.6), Delaware leads in pancreatic cancer (15.1/100,000), Maine in bladder cancer (12.4/100,000), and Montana in brain cancer (7.3/100,000). Florida presents a distinct profile among high language-isolation states, with notably lower rates in several categories compared to similarly language-isolated states.

### Relationship between language isolation and cancer risk

#### Correlation analysis

**Fig. 3:**
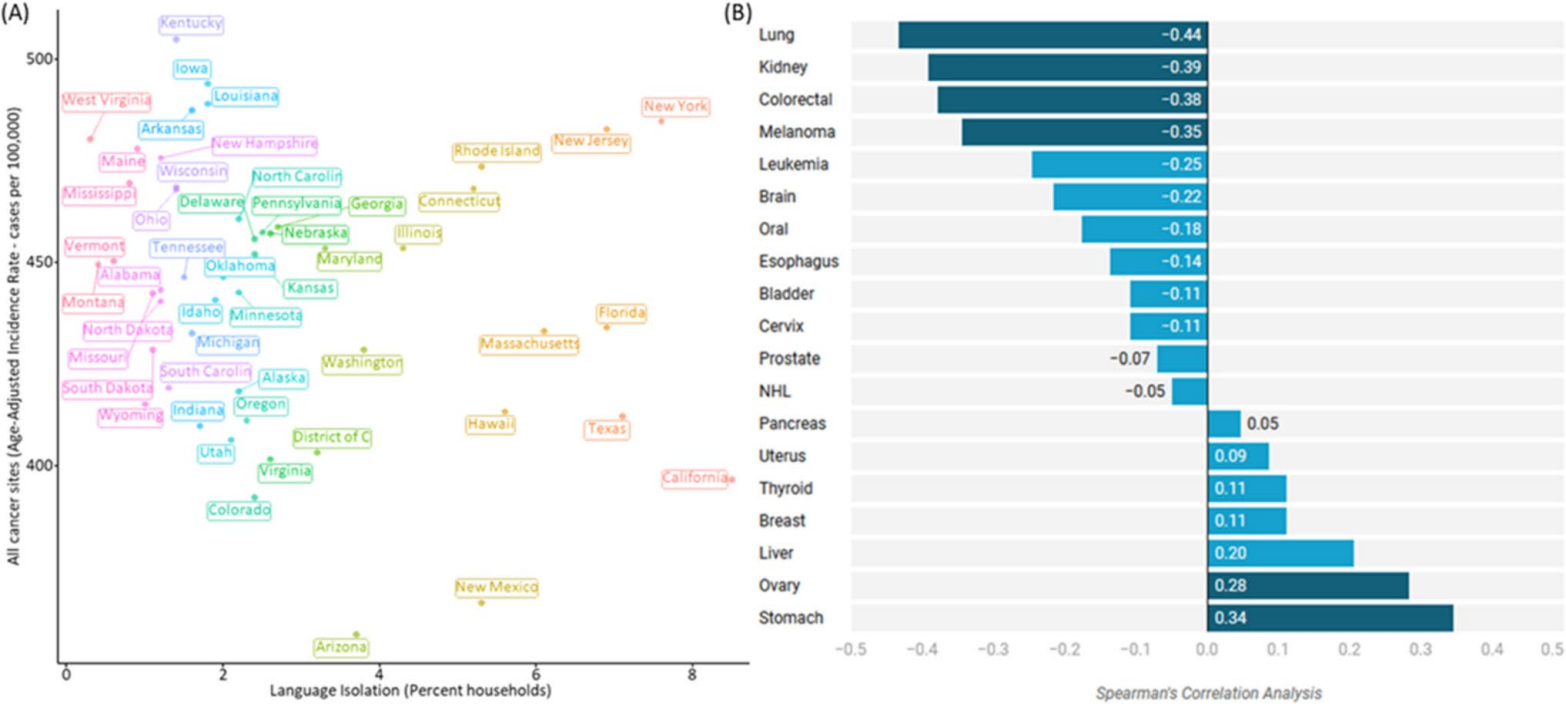
The association between non-English language isolation and cancer risk. **A** The relationship between the prevalence of language isolation and the age-adjusted cancer incidence rate per 100,000 cases across different states. The states with the highest language isolation rates, such as California, New York, Texas, New Jersey, and Florida, tend to have higher cancer incidence rates. **B** Spearman’s correlation coefficient shows the relationship between the prevalence of non-English language householders and the incidence rates of various cancer types across the United States

Our analysis revealed complex associations between language isolation and cancer incidence rates across the United States. States with both high cancer incidence and substantial language-isolated populations showed distinct patterns, particularly evident in California, New York, Texas, and New Jersey (Fig. 3A).

Supplementary Table S2 presents Spearman's correlation coefficients between the percentage of non-English language householders and the incidence rates of various cancer types across the United States. Statistical analysis demonstrated varied correlations between language isolation and different cancer types, with correlation coefficients ranging from − 0.435 to 0.344. Two cancer types showed significant positive correlations with language isolation: ovarian (r = 0.282, *p* = 0.045) and stomach (r = 0.344, *p* = 0.014) cancers. In contrast, four cancer types exhibited significant negative correlations: lung (r =  − 0.435, *p* = 0.001), kidney (r =  − 0.392, *p* = 0.004), melanoma (r =  − 0.346, *p* = 0.013), and colorectal (r =  − 0.380, *p* = 0.006) cancers (Fig. 3B).

#### State-specific patterns in language isolation and cancer incidence

**Fig. 4:**
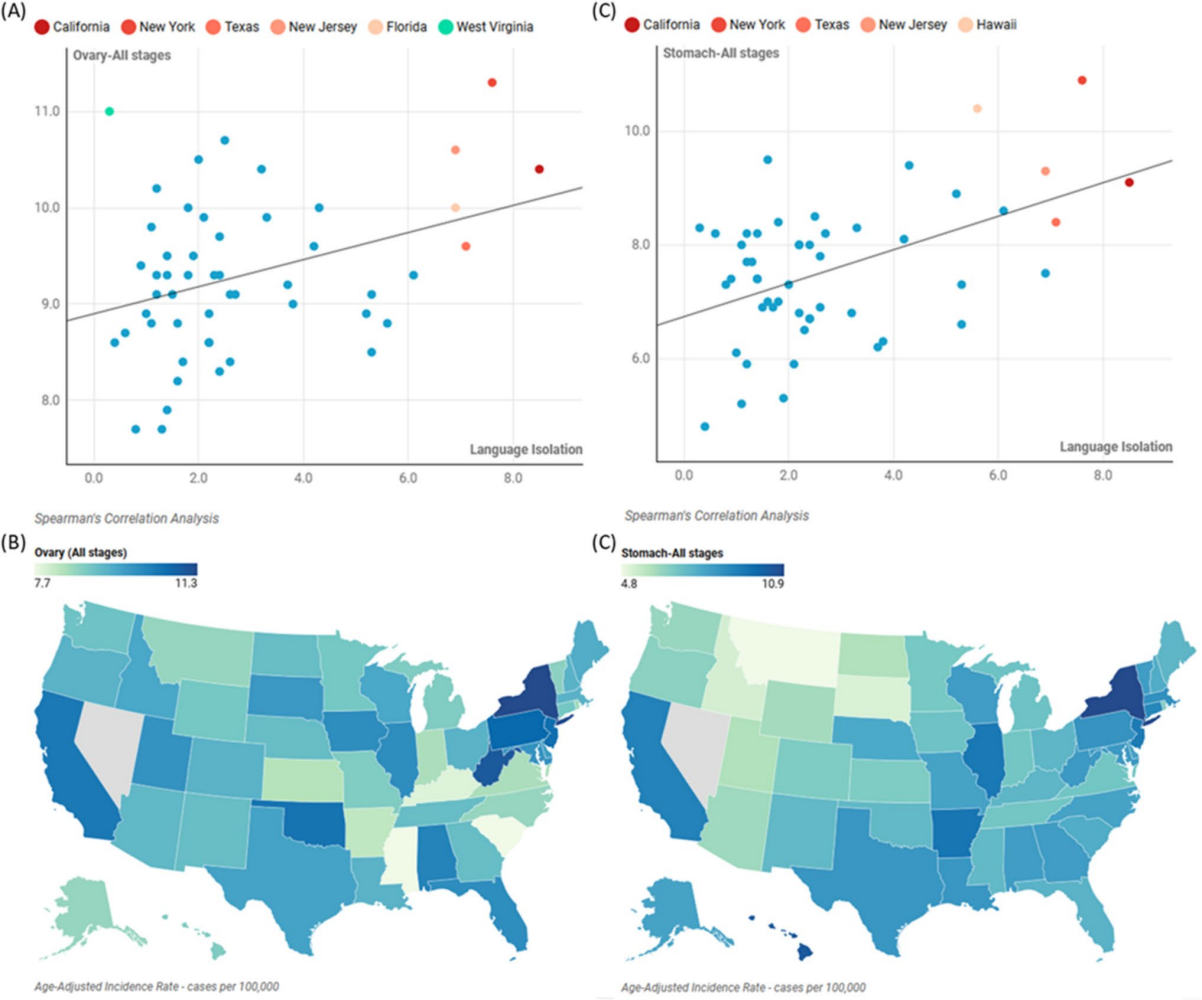
Correlation of the prevalence of language-isolated households with ovarian and stomach cancers across the United States. **A** 2D plot for the prevalence of language isolation (x-axis) and age-adjusted incidence rate of ovarian cancer per 100,000 cases (y-axis). Each dot represents a state. **B** US map showing the incidence rate of ovarian cancer across the United States. **C** 2D plot for the prevalence of language isolation (x-axis) and age-adjusted incidence rate of gastric cancer per 100,000 cases (y-axis). Each dot represents a state. **D** US map showing the incidence rate of gastric cancer across the United States

States with high language isolation rates demonstrated consistent patterns in certain cancers. California, with the highest language isolation rate (8.5%), showed elevated rates of ovarian (10.4 per 100,000) and stomach (9.1 per 100,000) cancers. Similarly, New York, ranking second in language isolation (7.6%), displayed high rates for both ovarian (11.3 per 100,000) and stomach (10.9 per 100,000) cancers, Fig. 4.

##### Florida: an exception to the pattern

Florida emerged as a significant outlier among states with high language isolation rates. Despite its 6.9% language isolation rate, Florida's cancer profile diverged markedly from other high language-isolation states across multiple cancer types. In gastrointestinal cancers, Florida (7.5 per 100,000) showed significantly lower stomach cancer rates compared to California (9.1), New York (10.9), and Texas (8.4). Gender-specific cancers also showed notable differences, with prostate cancer rates in Florida (88.2 per 100,000) being substantially lower than New York (138.5), Texas (105.1), and California (97.9), while breast cancer rates (123.0 per 100,000) remained consistent with other high language-isolation states. In screening-dependent cancers, Florida showed higher cervical cancer rates (9.0 per 100,000) compared to California (7.1) and New York (7.5).

### Late-stage cancer analysis

#### National distribution of late-stage cancer incidence

Late-stage cancer incidence rates vary significantly across cancer types nationally. Breast cancer shows the highest prevalence (41.6 cases per 100,000), followed by lung cancer (37.5 cases per 100,000), prostate cancer (23.1 cases per 100,000), and colorectal cancer (21.8 cases per 100,000). Brain cancer exhibits the lowest late-stage incidence rate nationally at 0.9 cases per 100,000 individuals., Supplementary Table S3.

#### Geographic distribution by cancer type

##### Lung cancer

Kentucky demonstrates the highest late-stage lung cancer incidence (61.9 cases per 100,000), followed by West Virginia (58.3 cases per 100,000) and Arkansas (56.2 cases per 100,000). In contrast, Utah (24.1 cases per 100,000), Hawaii (26.9 cases per 100,000), and Colorado (28.3 cases per 100,000) report the lowest rates.

##### Breast cancer

Georgia leads in late-stage breast cancer incidence (46.7 cases per 100,000), with Louisiana (45.9 cases per 100,000) and Alabama (45.1 cases per 100,000) following closely. Arizona reports the lowest rates (35.2 cases per 100,000), followed by Vermont (35.9 cases per 100,000) and New Hampshire (36.1 cases per 100,000).

##### Prostate cancer

The District of Columbia shows the highest late-stage prostate cancer incidence (32.6 cases per 100,000), followed by Louisiana (29.8 cases per 100,000) and Georgia (28.7 cases per 100,000). Vermont (15.2 cases per 100,000), Maine (16.1 cases per 100,000), and New Hampshire (16.4 cases per 100,000) report the lowest rates.

##### Colorectal cancer

Mississippi reports the highest late-stage colorectal cancer incidence (29.5 cases per 100,000), with Louisiana (28.9 cases per 100,000) and Arkansas (28.2 cases per 100,000) following. The lowest rates appear in Arizona (17.9 cases per 100,000), New Mexico (18.3 cases per 100,000), and Colorado (18.5 cases per 100,000).

#### Language isolation and late-stage cancer correlation

##### Negative correlations

Several cancer types show significant negative correlations with language isolation, including bladder cancer (r = -0.321, *p* = 0.022), colorectal cancer (r =  − 0.389, *p* = 0.005), kidney cancer (r =  − 0.368, *p* = 0.008), lung cancer (r =  − 0.480, *p* < 0.001), melanoma (r =  − 0.489, *p* < 0.001), and oral cancer (r =  − 0.334, *p* = 0.017), Supplementary Table S4.

##### Positive correlations

Other cancers demonstrate significant positive correlations with language isolation: liver cancer (r = 0.477, *p* < 0.001), stomach cancer (r = 0.486, *p* < 0.001), ovarian cancer (r = 0.276, *p* = 0.05), thyroid cancer (r = 0.408, *p* = 0.003), and uterine cancer (r = 0.561, *p* < 0.001).

##### No significant

Correlations Brain, breast, cervix, NHL, pancreas, and prostate cancers show no significant correlations with language isolation rates.

## Discussion

Our study reveals complex relationships between language isolation and cancer incidence rates across the United States, with notable geographic and cancer-type variations. States with high language isolation rates, particularly California, New York, Texas, and New Jersey, demonstrate elevated cancer incidence rates, though this relationship varies significantly by cancer type. The observed patterns suggest that language isolation's impact on cancer risk is heterogeneous and likely mediated by multiple factors.

The association between language isolation and cancer incidence appears to be influenced by several interconnected factors. Language barriers can create significant healthcare disparities through reduced access, diminished health literacy, and decreased utilization of cancer screening services [16]. This is evidenced by comparative studies between Spanish-speaking and English-speaking Hispanic populations, which found that despite similar employment rates, Spanish-speaking individuals had substantially higher rates of being uninsured (50% versus 20%) [17]. Such disparities in healthcare access frequently result in adverse health outcomes, both short-term and long-term [17, 18].

Healthcare communication challenges present particular concerns in cancer care. Language barriers often result in reduced patient engagement and diminished participation in shared decision-making [6, 16, 17]. The National Assessment of Adult Literacy data indicates that health literacy varies significantly based on primary language, with implications for understanding disease processes and treatment adherence [18–20]. Patients with limited health literacy often demonstrate reduced comprehension of medical conditions and treatment protocols, leading to decreased compliance and increased likelihood of missing follow-up appointments [19].

Cultural and genetic factors emerge as significant variables in the language isolation-cancer risk relationship [18, 19]. Specific dietary patterns and environmental exposures prevalent in certain language-isolated communities may contribute to observed cancer incidence variations [20]. Cultural values and beliefs can influence healthcare-seeking behaviors, particularly for gender-specific cancers. For instance, cultural attitudes regarding modesty may affect screening rates for breast and cervical cancers [21, 22]. Additionally, genetic factors may explain some observed patterns, such as the lower melanoma incidence in certain non-English speaking populations, potentially due to protective genetic factors like increased skin pigmentation, which can offer natural protection against the harmful effects of ultraviolet (UV) radiation [23, 24].

Socioeconomic factors further compound these relationships. Language-isolated communities often face economic challenges that limit access to healthcare services and preventive care [25, 26]. Our analysis reveals significant geographic disparities in cancer incidence, with striking variations in late-stage cancer diagnoses. States such as Kentucky, West Virginia, and Arkansas show concerning rates of late-stage lung cancer, while Utah, Hawaii, and Colorado demonstrate notably lower incidence rates.

The state-specific analysis reveals notable patterns. Kentucky's high late-stage lung cancer rate (61.9 per 100,000) represents a concerning public health issue, particularly when compared to Utah's significantly lower rate (24.1 per 100,000). Similar geographic variations exist for other cancers, with distinct regional patterns emerging for breast, prostate, and colorectal cancers. These variations likely reflect complex interactions between healthcare access, socioeconomic factors, and regional behavioral patterns.

The distinct cancer profile observed in Florida warrants particular attention. Despite having a high language isolation rate (6.9%), Florida demonstrates notably different cancer patterns compared to states with similar language isolation levels. This deviation is particularly evident in gastrointestinal and gender-specific cancers, where Florida reports significantly lower rates than California, New York, and Texas. Several factors may explain this exceptional pattern. First, Florida's unique demographic composition, particularly its large elderly population (20.9% aged 65 +), may influence cancer screening practices and detection rates. Second, the state's seasonal population fluctuations could affect healthcare delivery patterns and cancer surveillance data. Third, the marked urban–rural divide in healthcare access may contribute to these differences. The Florida case illustrates that language isolation alone may not determine cancer outcomes and highlights the importance of considering multiple demographic and healthcare system factors in understanding cancer risk patterns.

Study limitations warrant consideration. The cross-sectional design precludes definitive conclusions about temporal relationships between language isolation and cancer incidence [27]. Self-reported language data may introduce measurement bias [28], and the ecological nature of the study limits causal inference at the individual level [28]. Additionally, state-level aggregation may mask important local variations in language isolation, demographics, and healthcare access [25, 29].

These findings have important implications for public health policy and clinical practice. Future interventions should prioritize culturally sensitive health education programs and expanded access to linguistically appropriate cancer screening services [30, 31]. Healthcare providers and policymakers in high-risk areas should focus on overcoming language barriers through enhanced language assistance services [32], culturally relevant educational materials [33], patient navigation programs [16, 34], and improved physician–patient language concordance [35].

## Conclusions

In conclusion, addressing language isolation and related communication barriers is a critical step toward reducing cancer risk and improving health outcomes in linguistically diverse communities. By focusing on the unique challenges faced by these populations, we can make strides toward reducing disparities and improving cancer outcomes for these vulnerable communities. Future research should aim to understand the role of language barriers, cultural factors, and other social determinants of health in shaping cancer risk among language-isolated populations, ultimately informing the development of targeted interventions to reduce cancer disparities in these communities. By targeting the unique challenges faced by linguistically isolated populations in hotspot states, we can work towards mitigating disparities and enhancing cancer outcomes for these vulnerable communities.

## Supplementary Information

Below is the link to the electronic supplementary material.

Supplementary file1 (PDF 256 KB)

## Data Availability

Publicly available datasets were analyzed in this study. This data can be found here: (https://seer.cancer.gov/ (accessed on 29 January 2023)).
