# Supplementary material for: Unraveling the link between language barriers and cancer risk
Source: Cancer Causes Control. 2024 Dec 11;36(4):399–407. doi: 10.1007/s10552-024-01946-5 (PMC11982091; doi:10.1007/s10552-024-01946-5)
Supplement: Supplementary file 1 — Supplementary file1 (PDF 256 KB) [file 10552_2024_1946_MOESM1_ESM.pdf]

Table S1. Estimated incidence rates of cancer (all stages) and prevalence of language barrier households across the United States.

State	Percent	Age-Adjusted Incidence Rate - cases per 100,000																			
**State**	**Language Isolation**	**All cancer**	**Bladder**	**Brain**	**Breast**	**Cervix**	**Colorectal**	**Esophagus**	**Kidney**	**Leukemia**	**Liver**	**Lung**	**Melanoma**	**NHL**	**Oral**	**Ovary**	**Pancreas**	**Prostate**	**Stomach**	**Thyroid**	**Uterus**
United States	4.2	438.6	8	5.1	129.7	7.5	31.8	1.8	11.8	10.3	4.8	48.1	18.2	15	6.4	9.6	11.6	111.6	8.1	7.1	27.7
Alabama	1.2	443.4	7.5	5.2	127.2	10.1	35.8	1.3	12.4	9.6	4.9	47.4	16.2	12.9	7	10.2	11.1	118.2	8.2	4.6	21.3
Alaska	2.2	418.5	5.8	5.2	131.3	5.1	35.6	15	14.4	9.6	5.7	51	15.5	17.5	8.9	8.6	12.2	97.8	8	6.8	28.2
Arizona	3.7	358.7	7.2	4.9	112.9	5.8	26	1.2	9.7	8.3	3.7	37.1	19.7	10	5	9.2	10.6	68.7	6.2	6.2	23.3
Arkansas	1.6	487.6	8	5	128.8	9.5	38.8	1.3	17.7	10.5	5.1	62	21.1	15.1	6.9	8.2	10.9	112.9	9.5	6.7	26.5
California	8.5	396.8	6.2	4.6	124.7	7.1	29.7	1.5	10.3	8.9	5.7	34.1	17.8	14.7	5.6	10.4	11.1	97.9	9.1	6.9	27.7
Colorado	2.4	392.4	7.9	5	131.1	5.6	28.1	1.5	10.6	9.4	4.4	37	16.5	13.2	5.5	9.3	11.1	94.3	6.7	8.5	22.7
Connecticut	5.2	468.3	9.1	6	142.5	5.2	27.2	2.2	11.5	11.4	3.9	52.9	14.8	17.1	8.1	8.9	11.7	132.4	8.9	9.1	29.3
Delaware	2.4	455.9	9.8	4.6	147.2	6.3	27.7		10.3	8.2	3.9	45.9	21	12.2	6.5	9.7	15.1	129.3	8	8	32.6
District of C	3.2	403.5	7.1		125.3	8.2	27.9		6.4	8.2	6.7	38.9	7.1	12.4	6	10.4	11.7	131.6	6.8		31.5
Florida	6.9	434.1	7.4	5.3	123	9	30.6	1.8	10.1	11.6	4.3	48	18.2	16.5	6.7	10	11.1	88.2	7.5	6.7	26.3
Georgia	2.7	458.9	7.4	4.7	129	8.2	33.2	1.9	12	11.3	4.9	48.9	20.3	13.8	6.8	9.1	11.4	139	8.2	6.3	24.4
Hawaii	5.6	413.5	5.4	2.7	140.5	7.4	32.2	1.7	9.3	7.6	5	36.3	19.3	11.3	7	8.8	11.1	104.2	10.4	6	35.7
Idaho	1.9	440.9	7.1	5.5	127.8	8	31.1	1.5	13.8	12.3	4.6	44	25.3	15.8	6.4	9.5	11.6	125.1	5.3	7.1	26.5
Illinois	4.3	453.6	8.2	4.7	134.7	7.4	33.4	2.1	12.5	9.7	4.9	52.7	20.3	15.5	6.5	10	12.4	114.2	9.4	7	30.2
Indiana	1.7	410	7.5	5.3	118.3	8.1	33.3	1.6	11.4	8.7	4.3	55.5	15.5	13.1	6.2	8.4	10.2	92.1	6.9	5.9	27.9
Iowa	1.8	494.1	8.4	5.1	138.1	7.5	36.3	1.9	13.6	11.7	4.1	54.4	30	17	9.1	10	11.6	128.2	7	8.8	31.7
Kansas	2.4	452.2	9.2	6.2	132.9	7.6	34.5	2.1	12.7	12.3	4.6	48	25.1	14.5	6.8	8.3	9.8	114.7	6.7	7.5	28.2
Kentucky	1.4	505	8.9	6.3	130.6	9.2	40.3	2.2	14.1	13.2	4.6	75.6	24.6	15.7	7.6	7.9	11.6	111	7.4	6.7	29.1
Louisiana	1.8	489.2	7.2	4.7	128.4	8.9	39.6	1.6	17.1	11.2	5.3	51.6	13.3	16.7	5.8	9.3	13.2	146.5	8.4	7.3	20.8
Maine	0.9	478.1	12.4	4.4	133.8	5.2	33.8	2.6	11.1	11.8	4.6	63.7	22.9	14.9	7.7	9.4	12.2	106.9	7.4	8	33.2
Maryland	3.3	453.6	7.5	5.2	139.2	6.6	33	1.9	11.7	9.2	5.1	47.5	20.3	13.6	5.9	9.9	12.3	141.3	8.3	6.6	28
Massachusetts	6.1	433.2	10.2	5.2	137.7	4.8	27.6	2.3	9.2	9.3	4.5	56.4	12.3	14.7	5.7	9.3	11.9	110.5	8.6	7.8	28.3
Michigan	1.6	432.7	8.9	5	123.9	6.2	31.8	2.3	10.7	10	4.1	53.8	16.5	15.3	6.4	8.8	12.6	116.4	7	6.5	28.3
Minnesota	2.2	442.8	8.6	4.9	134	5.5	29.7	2	11.6	11.1	4.3	47.6	32.6	16.2	6.5	8.9	12.1	108.1	6.8	6.3	29
Mississippi	0.8	469.6	7.5	5.2	126.7	9.1	39.9	2	16.1	10	4.7	54.6	16.1	13.7	6.4	7.7	12	141.1	7.3	4.5	23.7
Missouri	1.1	442.5	7.3	5.3	136.4	8.9	33.9	1.8	13.1	10.8	4.9	60.7	14.5	15.6	5.6	8.8	12.2	95.2	8	6.2	28.4
Montana	0.4	449.6	8.5	7.3	135.2	6.8	30.5	2.3	10.5	9.2	3.8	44.7	25.7	14	7.6	8.6	11.8	132.8	4.8	9.9	26.2
Nebraska	2.6	457.3	7.3	6.3	129.6	9.1	33.2	2.6	14.2	10.2	4.6	46.6	26.4	14.8	6.8	9.1	11.1	130.2	7.8	8.4	28.7
New Hampshire	1.2	475.8	11.7	6.1	139.6	6.1	34.5	2.3	12.3	10.3	4.2	58.7	22.4	18.8	6.9	9.3	12.5	120.7	7.7	5.3	30.7
New Jersey	6.9	482.9	9.8	5.6	142.6	6.9	34.3	1.7	10.9	11.5	4.5	47	16.9	16.9	6.2	10.6	13.9	145.4	9.3	9.3	32.1
New Mexico	5.3	366.5	6.2	4.2	124.3	7.8	25.4	1.6	10.8	7.9	4.5	29.4	12.9	11.1	5.1	9.1	10.7	86.1	7.3	9.8	25.6

New York	7.6	484.9	9.9	5.2	141.2	7.5	32.4	1.9	11.4	11.4	4.8	52.1	15.1	17.5	7.3	11.3	12.9	138.5	10.9	10.8	31.1
North Carolin	2.2	460.8	7.8	5.1	138.1	6.8	29.3	1.9	12.7	10.8	4.4	53.6	21.3	14.2	6.8	8.6	11.9	129.5	8	5.4	25.8
North Dakota	1.2	440.6	8	4.1	137.2	4.9	34.9		12.6	12.1		53.4	19.2	15.4	8.1	9.1	10.2	124.2	5.9	10.1	27.1
Ohio	1.4	468	9.4	6	131.2	8	32.7	2.1	12.7	9.6	4.6	58	22.5	15.9	6.8	9.3	11.9	118.9	7.4	7.3	31.6
Oklahoma	2	446.6	7.6	5.4	120.1	10.3	34.6	2.1	16.4	8.8	5.1	57.3	14.6	14	7.3	10.5	10.9	105.2	7.3	7.1	24.7
Oregon	2.3	411.3	9	4.2	134.7	6.2	28.2	2.1	9.8	10	4.7	43.8	21.6	13.9	6.5	9.3	10.6	96.5	6.5	5.5	30.2
Pennsylvania	2.5	457.5	9.7	5.7	130.6	7	34	1.9	11.3	10.7	4.6	52.3	16.7	15.8	6.7	10.7	12.2	113.3	8.5	9	32.5
Puerto Rico		367.8	4.9	3.2	102.1	12.1	32.2	0.9	6.5	8.6	4.1	11.1	3.9	11.9	4.9	7.1	7.6	156.1	8	10.6	32
Rhode Island	5.3	473.7	10.4	5.9	144.7	6	31	2.1	13.2	11.2	4.9	56.6	19.1	16.1	5.4	8.5	11.4	130.7	6.6	9.1	27.4
South Carolin	1.3	419.4	6.8	4.9	132	7.9	31	1.4	12.2	8.7	5.1	47.8	15.6	11.4	6	7.7	10.4	108.5	7.7	5.7	23.1
South Dakota	1.1	428.6	6.7	5.1	126.6	5.6	33.8		14.1	12.9	3.7	50.5	21.6	11.2	6	9.8	8.3	121.4	5.2	9.2	28.1
Tennessee	1.5	446.5	8.1	5.1	121.2	6.9	33.3	1.5	15.1	9.6	4.5	58.4	17.6	14.3	5.7	9.1	10.6	115	6.9	5.6	25.9
Texas	7.1	412.4	6	4.9	122.4	9.5	31.7	1.4	14.2	10.1	6.3	38.3	11.9	14.1	5.5	9.6	11.1	105.1	8.4	6.4	24.8
Utah	2.1	406.5	6.8	6.7	118.4	5.1	25.8		11.5	10.8	3.8	25	37.3	16.1	6.1	9.9	10.7	115.3	5.9	9	25.4
Vermont	0.6	450.5	9.1	5.8	139.2	8.2	22.3		11.3	10.6		50.1	32.5	16.2	5.2	8.7	12.2	108.5	8.2	6.9	27
Virginia	2.6	401.8	7.7	4.5	128.2	6.1	29.8	1.6	11.1	8.5	4	45.2	17	13.3	5.8	8.4	10.7	98.8	6.9	5.3	25.4
Washington	3.8	428.6	8.4	5.4	132.5	6.6	28.1	2.2	10.3	10.5	5.4	46.7	22.2	15.4	7.6	9	11.6	100.5	6.3	5.7	26.6
West Virginia	0.3	480.5	7.9	5.4	127	7.4	38.2	1.7	13.8	11.7	4.8	65.3	21.1	16.3	5.2	11	11.2	100.4	8.3	7.6	38.5
Wisconsin	1.4	468.7	9.6	5.2	138	6	30.9	2.5	11.3	13.1	3.8	52.4	20.1	17.4	7.2	9.5	12.5	125.9	8.2	7.5	29.9
Wyoming	1	415.3	8.6	6.2	114.9	10.2	29.9		12.3	11.5		45.7	24.5	15.1	7.3	8.9	7.3	112.1	6.1	8.1	20.8

Table S2. Spearman's correlation coefficient between the percentage of non-English language householders and incidence rates of cancer (All stages).

**Cancer**	**Correlation Coefficient**	***p*-value**
Bladder	-0.110	0.44
Brain	-0.217	0.13
Breast	0.110	0.44
Cervix	-0.109	0.45
Colorectal	-0.380	**0.006**
Esophagus	-0.137	0.38
Kidney	-0.392	**0.004**
Leukemia	-0.247	0.08
Liver	0.204	0.16
Lung	-0.435	**0.001**
Melanoma	-0.346	**0.013**
NHL	-0.051	0.72
Oral	-0.178	0.21
Ovary	0.282	**0.045**
Pancreas	0.045	0.75
Prostate	-0.072	0.61
Stomach	0.344	**0.014**
Thyroid	0.109	0.45
Uterus	0.085	0.55
All_cancer	-0.245	0.08

Bold values indicate significance at $p < 0.05$.

Table S3. Geographical Distribution of Late-Stage Cancer Incidence Rates in the United States.

State	Percent	Age-Adjusted Incidence Rate - cases per 100,000																	
**State**	**Language Isolation**	**Bladder**	**Brain**	**Breast**	**Cervix**	**Colorectal**	**Esophagus**	**Kidney**	**Liver**	**Lung**	**Melanoma**	**NHL**	**Oral**	**Ovary**	**Pancreas**	**Prostate**	**Stomach**	**Thyroid**	**Uterus**
United States	4.2	2.3	0.9	41.6	3.6	21.8	3.1	4.8	3.6	37.5	3.1	12.1	7.4	7.3	9.8	23.1	3.6	4.3	7.2
Alabama	1.2	2.1	0.6	39.6	3.9	23.1	2.7	4.5	2.6	39.5	3.1	10	6.8	6.8	8.7	19.1	3.3	2.2	4.8
Alaska	2.2	2.3	0.9	41.6	3.1	24.5	3.9	5.4	4	36.5	2.1	11	7.4	6.4	9.1	22.8	4.8	3.7	6.7
Arizona	3.7	1.8	0.6	35.2	2.6	17.9	2.3	4.3	2.8	26.5	2.9	9.2	5.3	6.7	8.2	17.3	2.7	4	5.9
Arkansas	1.6	2.2	0.8	40.3	4.4	26.3	2.7	5.5	3.7	49.6	3.2	11.2	8.9	7.1	9.3	20.7	3.5	3	6.2
California	8.5	2.1	1	41.4	3.7	19.7	2.3	4.5	3.9	26.8	2.9	11.9	6.5	8	9.2	23.6	4.4	4.9	8
Colorado	2.4	2	1	42.3	2.6	19.1	2.8	3.9	2.8	25.5	3.7	11.8	6.5	6.8	8.5	23.1	3.1	5.1	5.8
Connecticut	5.2	2.7	0.8	41	2.6	18.8	3.4	4.8	3.8	37.3	2.9	13.4	7.6	7.8	11.1	27.6	4.3	5.8	8.7
Delaware	2.4	2.2	1	43.2	3.1	20.9	3.3	4.4	3.8	41	3.9	12.4	7.7	6.7	10.6	21.5	3.7	4.4	8
District of Columbia	3.2	2.5		46.2	3.4	18.5	2	3.1	4.2	27.2	1.5	9.3	6.7	8.3	10.6	28.6	3.5	3.4	9.6
Florida	6.9	2.3	1	41.4	4.4	21.1	2.8	4.2	3.3	36.7	3.5	11.4	8.4	7.4	9.5	18.8	3.3	3.9	7.4
Georgia	2.7	2.2	0.9	46.7	4.1	22.5	2.9	5.2	4.6	42.5	3	11.9	8.6	7.9	10.5	23.4	3.6	3.2	7.8
Hawaii	5.6	1.8	0.6	40.8	2.8	21.8	2.6	4.1	4.4	31.8	2.3	9.2	7.6	6.7	9.9	26.9	4.3	5.3	9.6
Idaho	1.9	2.9	1.1	44.4	3.3	22	3.4	4.9	3.5	33	3.7	13.1	6.9	8	10.3	24.7	3	5.1	6.8
Illinois	4.3	2.6	0.8	44.6	3.5	24.2	3.3	5.3	3.4	41.3	3.1	12.6	7.8	7.5	10.7	26.3	4.2	4.7	8
Indiana	1.7	2.8	0.9	40	4.3	24.7	4.1	5.5	3.4	48.4	4	12.5	8.1	7.3	10.4	23.1	3.4	3.4	6.9
Iowa	1.8	2.6	1.2	43.1	3.6	24.1	4.2	6.1	3.7	45.1	4	14.7	8.3	7.5	10.7	30.4	2.9	4.7	6.7
Kansas	2.4	2.2	0.7	41.7	4.1	22.5	3.3	5.1	3	37.2	3.1	12.9	7.1	7	9.1	20.6	2.9	3.7	6.6
Kentucky	1.4	2.7	1.2	41.6	5.1	25.6	3.6	5.9	4.1	61.9	3.9	13.5	9.2	6.7	10.2	20.7	3.4	4.1	6.9
Louisiana	1.8	2.2	1	46.8	4.8	25.6	2.9	6.2	5.2	46.2	2.5	12.3	8.3	6.4	11.2	28.1	3.8	3.4	6.3
Maine	0.9	3.2	1.2	38.9	2.2	20.5	5.1	5.1	2.6	48.4	3.3	14.2	8.9	5.8	9.4	24.7	3.1	4.7	6
Maryland	3.3	2.3	0.9	44.2	3.1	20.5	2.9	4.1	3.5	34.8	2.5	12.2	7	7.1	10	21.8	3.6	4.3	7.8
Massachusetts	6.1	2.7	1.5	36.2	2.5	18.6	3.8	4.4	3.7	37.4	3.1	12.7	7.8	7.3	10.4	26.1	4	5.3	7.4
Michigan	1.6	2.5	1.1	40.3	3.1	21.5	3.6	4.6	2.9	42.1	3.3	13	7.6	6.9	10.2	23.1	3.3	3.7	7.6
Minnesota	2.2	2.5	1.2	41.4	2.1	20.9	3.5	5.4	3.4	34.5	3.4	14.1	6.6	6.7	9.6	29.1	3.1	4.3	6.7
Mississippi	0.8	1.9	0.7	46.1	4.9	29.5	3.1	5.7	4	51.1	3.1	10.7	8.2	5.8	10.7	25.4	3.8	2.3	6.5
Missouri	1.1	2.5	0.9	43.7	4.1	24	3.6	5.5	3.5	46.1	3.2	12.6	8.4	6.5	9.7	22.3	3.3	3.7	6.8
Montana	0.4	2.8	0.7	43	3.1	21.5	3.5	5	2.7	32.9	3.6	11.7	7.3	7.2	9.3	28.4	3.3	4	5.7
Nebraska	2.6	2.3	1	42	3.2	23.8	3.3	5.4	2.5	35.5	3.8	13.2	7.3	6.2	9.7	27.6	3	4.8	6.8
New Hampshire	1.2	3.3	1.4	41.7	2.2	20.9	4.6	4.6	2.4	42	4.1	12.9	7.3	6.8	9.7	24.7	2.8	4.1	6.1
New Jersey	6.9	2.4	0.8	43.7	4	22.6	2.8	4.7	3.7	35.3	2.7	13.3	7.1	8.2	11.1	22.5	4.3	5.7	8.6
New Mexico	5.3	1.8	0.5	37.8	3.6	18.2	2	5.4	3.3	21.5	2.4	7.8	5.5	7.3	7.3	17.9	3.3	5.7	7.3

New York	7.6	2.4	0.9	42.1	3.7	21.2	3.1	4.6	4.3	37.3	2.5	13.1	7.2	8.2	11.3	25.6	4.7	6.4	9.1
North Carolina	2.2	2.3	0.8	44.4	3.4	21.9	3	4.7	4	44.9	3.1	12.2	8.3	6.8	10.2	24	3.6	3.5	6.8
North Dakota	1.2	2.3	1.2	43.6	2.9	24	3.7	4.7	2.8	38.6	3.4	12.5	6.8	6.8	8.8	27.2	3	4.8	6.4
Ohio	1.4	2.7	0.8	43.7	3.8	23.9	3.9	5.3	3.1	44.8	3.9	13.3	8.2	6.9	10	24.3	3.3	3.8	7.6
Oklahoma	2	2.3	0.8	43	4.9	22.7	2.9	5.7	3.3	42.5	3.3	11.1	7.4	7.5	8.4	18.3	3	2.9	6.3
Oregon	2.3	2.6	1.2	41.1	2.5	20.1	3.5	4.3	3.5	32.9	2.8	11.9	8	7.9	9.5	25.1	3	5	6.5
Pennsylvania	2.5	2.9	0.9	42.8	3.5	24.2	3.9	4.7	3.7	41.4	3.5	13.8	7.9	8	11.4	24.2	3.7	4.8	8.1
Puerto Rico		0.8	0.4	34.6	4.1	19.1	1.1	2.1	2.1	7.9	0.6	6.3	4.6	4.9	5.5	12.8	3.2	5.8	6.6
Rhode Island	5.3	2.7	1.1	39.6	2.8	19.4	3.5	5.2	3.8	43.6	3.7	12.2	7.7	6.4	10.2	25	3.8	5.2	6.7
South Carolina	1.3	2.1	0.8	43.8	3.8	21.4	3	4.6	3.3	40.1	2.9	10.2	8	5.8	10	20.7	3.6	2.7	6
South Dakota	1.1	2.3	0.9	37.6	3.2	23.8	3.8	4.9	3	38.1	3.1	13.2	6.6	7.9	9.8	26	2.7	5.3	5.6
Tennessee	1.5	2.4	0.8	40.5	3.8	23.9	3.1	5.1	3.5	48.6	3.4	10.9	8.4	6.7	8.9	22.2	3	3.2	6.2
Texas	7.1	1.7	0.8	37.8	4.2	21.8	2.3	5.5	4.4	31.1	2.6	10.2	6.6	6.9	8.7	20.7	3.6	3.8	6.5
Utah	2.1	1.8	1.2	41.5	2.6	16.2	2.1	4.2	3	18.3	4.7	12.5	5.8	8.2	8.4	25.2	2.9	5.3	6.6
Vermont	0.6	2.7	1.3	37.3	1.9	19.4	3.7	4.8	2.3	39.9	3.8	12.6	8.5	6.7	9	29.1	3.2	5	6.5
Virginia	2.6	2.4	0.8	40.8	2.6	19.9	2.8	3.9	3	35.8	2.9	10.8	6.8	6.6	8.8	19.5	3.3	3.6	6.4
Washington	3.8	2.4	1.1	41.5	2.7	20	3.3	4.6	3.6	33.3	3.1	12.1	7.2	7.4	10	25.3	3.2	4.4	6.6
West Virginia	0.3	2.9	1	41.3	4.3	25.3	4.3	5.8	3.3	55.1	3.6	13.9	9.6	8.1	9.5	16.8	2.9	3.4	6.9
Wisconsin	1.4	2.7	1.1	43.1	2.9	21.4	4.1	5.5	3.4	40.5	3.5	14.8	7.9	7.8	10.7	30.8	3.7	4.5	8
Wyoming	1	2.6	1	37.8	3.5	20.2	2.4	4.9	2.2	26.5	3.8	9.9	5.9	7.5	8.2	22	2.3	4.2	5.1

Supplementary Table S4. Correlation Between Non-English Speaking Households and Late-Stage Cancer Incidence Rates in the United States.

Cancer	Correlation Coefficient	p-value
Bladder	-0.321	**0.022**
Brain	-0.160	0.27
Breast	-0.043	0.77
Cervix	-0.060	0.68
Colorectal	-0.389	**0.005**
Esophagus	-0.471	**<0.001**
Kidney	-0.368	**0.008**
Liver	0.477	**<0.001**
Lung	-0.480	**<0.001**
Melanoma	-0.489	**<0.001**
NHL	-0.183	0.20
Oral	-0.334	**0.017**
Ovary	0.276	0.05
Pancreas	0.171	0.23
Prostate	-0.058	0.68
Stomach	0.486	**<0.001**
Thyroid	0.408	**0.003**
Uterus	0.561	**<0.001**

Bold values indicate significance at $p < 0.05$.
